# A Rare Forehead Mass: The Chondroid Syringoma

**DOI:** 10.7759/cureus.5763

**Published:** 2019-09-25

**Authors:** Khuram Khan, Anant Dinesh, Marina Landa, Ryan Engdahl

**Affiliations:** 1 Surgery, Columbia University College of Physicians and Surgeons at Harlem Hospital Center, New York, USA; 2 Pathology, Columbia University College of Physicians and Surgeons at Harlem Hospital Center, New York, USA; 3 Plastic Surgery, Columbia University College of Physicians and Surgeons at Harlem Hospital Center, New York, USA

**Keywords:** chondroid syringoma, neoplasm, cutaneous nodule, head and neck

## Abstract

The chondroid syringoma is an extremely rare skin tumor most commonly found in the area of the head and neck region. Its rarity, potential for malignancy, and frequent misdiagnosis for other more common tumors can impart unique challenges in diagnosis and management. Diagnosis is usually revealed by excision followed by histologic examination. We report a case of a 42-year-old male with no prior medical history diagnosed with chondroid syringoma of the forehead and review the relevant literature.

## Introduction

The chondroid syringoma is a rare benign skin appendageal neoplasm first described by Hirsch and Helwig in 1961 [1]. It is typically a benign tumor that occurs most frequently in the head and neck region. Common sites include the scalp, cheek, nose, upper lip, chin, and the forehead. These tumors are unique because the incidence is very low <0.098% amongst all primary skin tumors [2]. Clinical presentation alone of this tumor is often insufficient to make the diagnosis, which occurs following histologic evaluation.

## Case presentation

**Figure 1:**
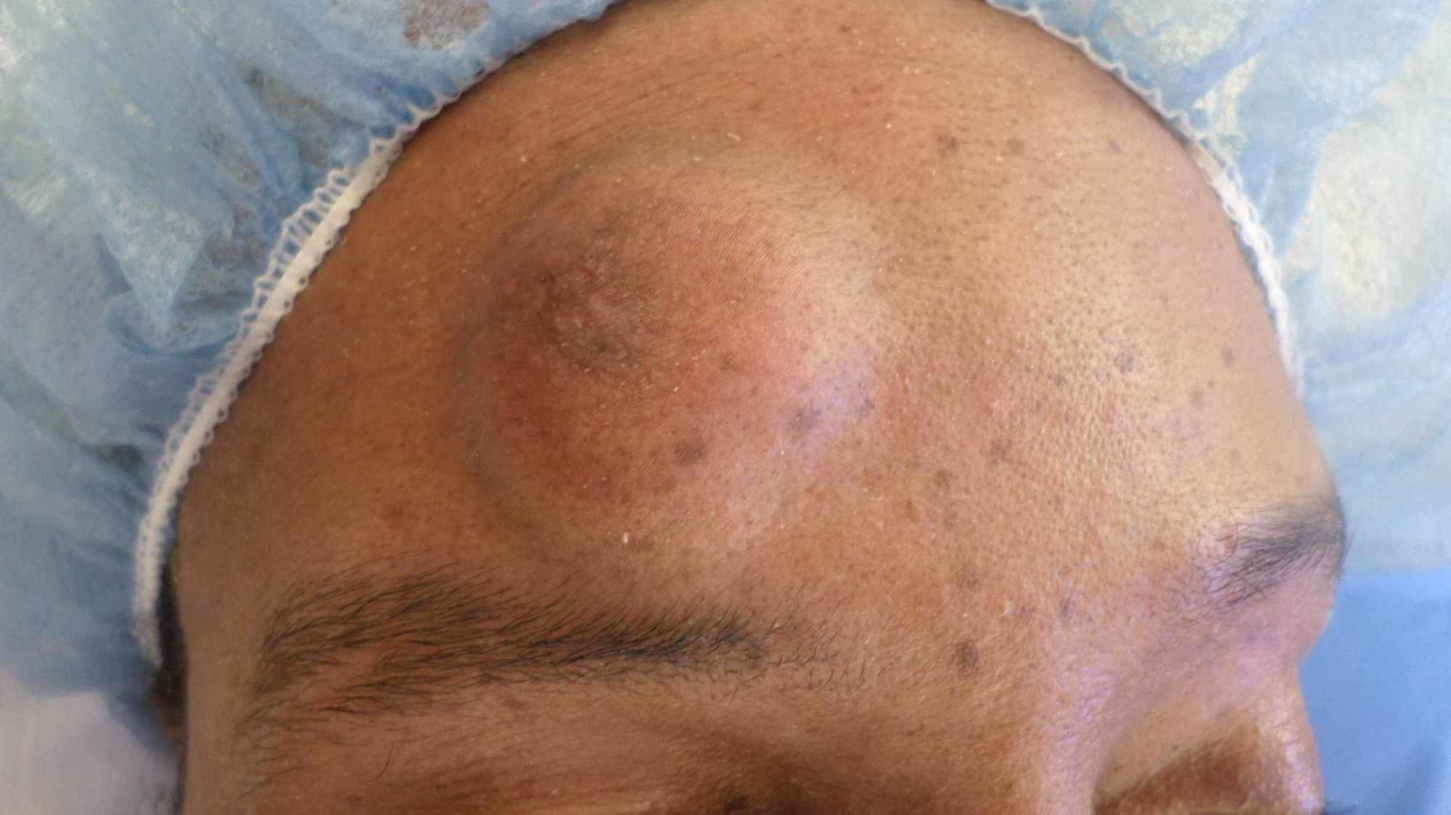
Forehead mass

**Figure 2:**
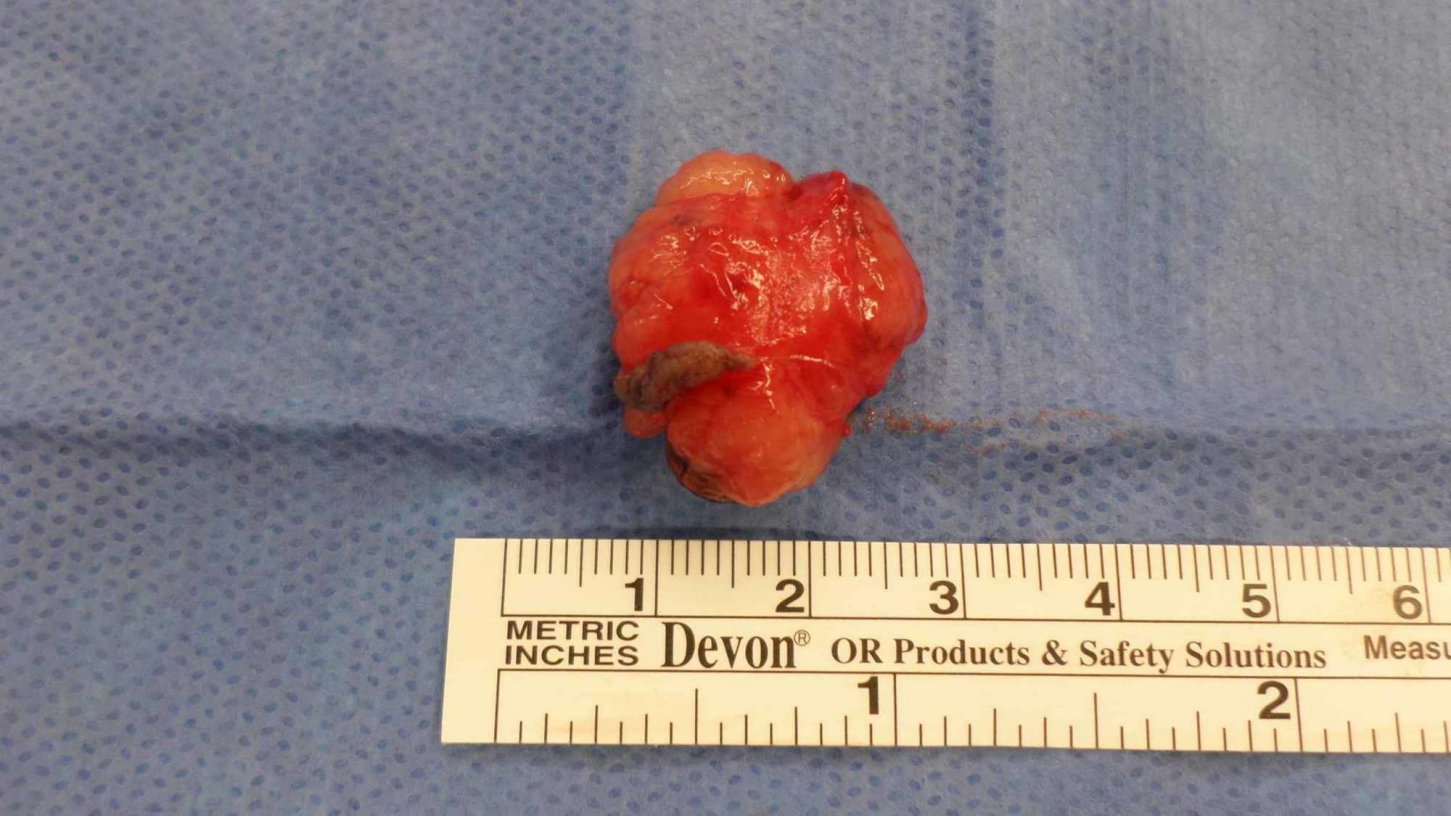
Excised forehead mass

**Figure 3:**
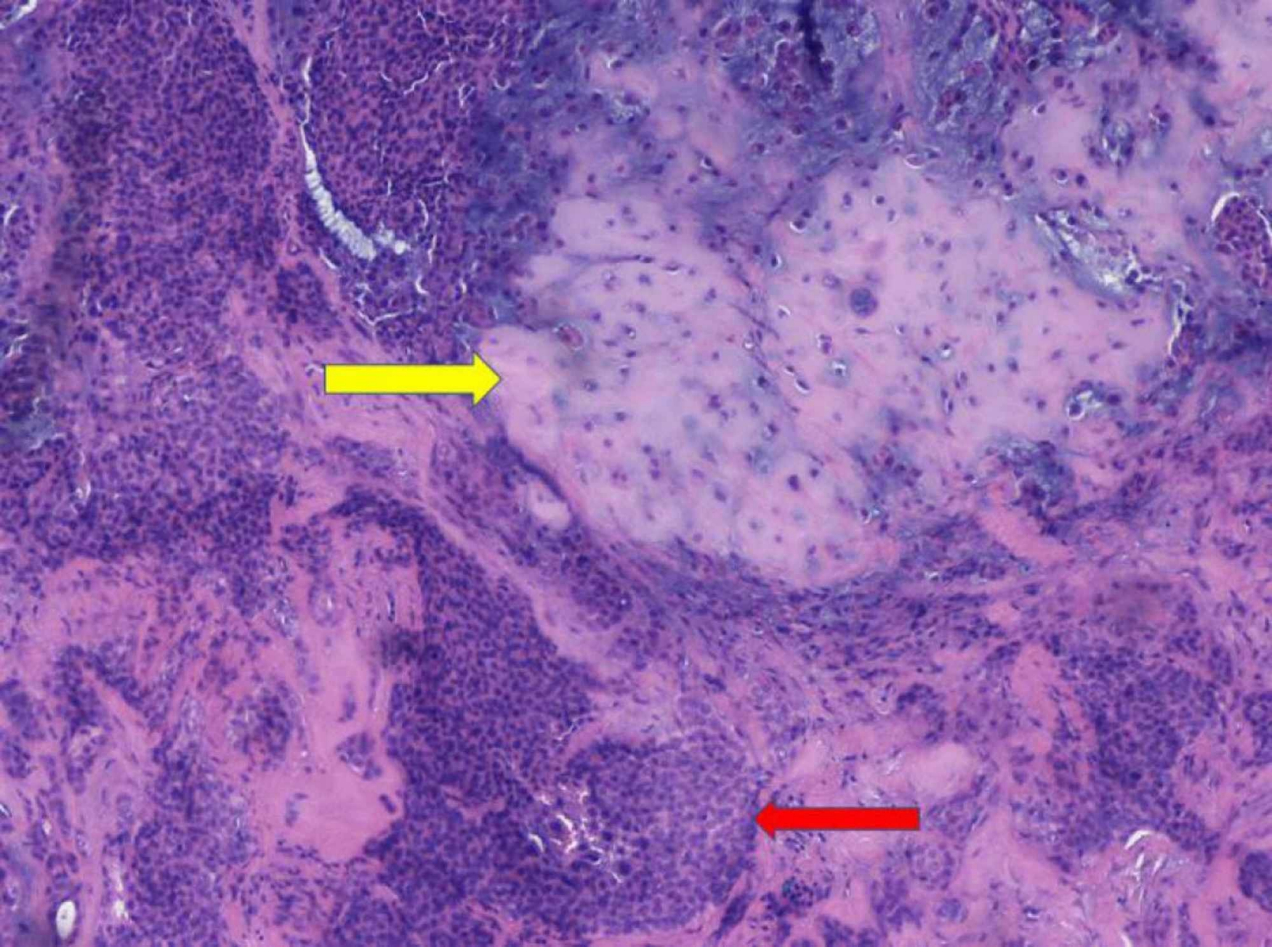
Histology demonstrating chondromyxoid stroma (yellow arrow). Cuboidal epithelial cells with pink eosinophilic cytoplasm in nested growth pattern (red arrow).

A 42-year-old man with no medical history presented with a firm, painless, mobile 2 cm nodule (Figure 1). He reported the lesion had been present for 12 months and had grown slowly during this time. No palpable other masses nor cervical lymphadenopathy was noted. He underwent operative excision of the mass. This revealed a firm fibro-fatty mass in the subcutaneous tissues that appeared well-circumscribed, firm, and white-tan in color (Figure 2). Histology of the mass revealed round tubules lined by cuboidal epithelial cells with pink eosinophilic cytoplasm and regular oval to round nuclei, with the tubules set in a chondroid stroma (Figure 3). Given these histopathological findings, a diagnosis of chondroid syringoma was made. The patient did well after the operation and there was no recurrence at follow-up visits.

## Discussion

Chondroid syringoma, or a cutaneous mixed tumor, is an adnexal neoplasm of either apocrine or eccrine origin. It presents as a solitary, firm, dermal or subcutaneous nodule, usually between 0.05-3 cm [2]. The lack of a distinctive clinical appearance often results in a misdiagnosis as another entity such as an epidermoid cyst, pilar cyst, or neurofibroma. It can occur in adults and children. They are more commonly found on the head, neck, and scalp and less frequently on the trunk, axilla, inguinal area, and genitalia. Typical history is a slow-growing, painless nodule. Rapid growth or ulceration should prompt concern for a malignancy, which is very rare in these tumors. Pathology typically reveals a well-circumscribed nodule seen within the deep dermis or subcutis [3]. The term Chondroid Syringoma by Hirsch and Helwig was in place of pleomorphic adenoma of the skin, as the tumor is epithelial in nature, with associated secondary changes in the stroma [1]. Chondroid, myxoid, fibrous, or even osseous stoma can be seen with epithelial structures typically apocrine or eccrine, although folliculosebaceous elements can be seen. Various treatment options have been proposed for chondroid syringoma, including excision, electrodessication, dermabrasion, and vaporization with argon or CO2 laser [2]. Because of the risk of malignancy, the first-line treatment is total excision of the tumor [2]. However, there are no standard recommendations for removal of these lesions. Following excision, follow-up should be done to look for local recurrence and any feature of malignancy. Recurrence of benign lesions has been reported for inadequately excised lesions of 18% [4]. Recurrent lesions can be treated by surgical re-excision [5]. Malignant cases have been reportedly mostly seen in women and more commonly in the extremities [6]. Tumors greater than 3 cm in size have a greater likelihood of malignancy [6]. Histological features that suggest malignancy include cytologic atypia, infiltrative margins, satellite tumor nodules, tumor necrosis, and involvement of deep structures. For malignant lesions the initial treatment modality is aggressive surgery. Adjuvant radiotherapy, with or without chemotherapy, may be recommended [6]. It is suggested that these myoepithelial carcinomas appear to be distinct and do not arise from preexisting benign chondroid syringoma [7]. Malignant lesions present with metastasis to lymph nodes or other sites such as bone or lung in around 50% patients and on pathology can be distinguished from benign with presence of cellular atypia [8]. Most of these lesions have been treated with wide local excision followed by adjuvant radiotherapy. No definite guidelines regarding management of these myoepithelial tumors are available due to rarity of the disease.

## Conclusions

If there is a nodule in the head and neck region, although rare, the chondroid syringoma should be considered in the differential diagnosis. With the low incidence of these cutaneous tumors along with non-prominent clinical presentation, excision of the mass followed by histopathological examination is recommended for diagnosis and treatment.
